# CO-Releasing Molecule-2 Induces Nrf2/ARE-Dependent Heme Oxygenase-1 Expression Suppressing TNF-α-Induced Pulmonary Inflammation

**DOI:** 10.3390/jcm8040436

**Published:** 2019-03-30

**Authors:** Chih-Chung Lin, Li-Der Hsiao, Rou-Ling Cho, Chuen-Mao Yang

**Affiliations:** 1 Department of Anesthetics, Chang Gung Memorial Hospital at Linkuo, Kwei-San, Tao-Yuan 333, Taiwan; chihchung@adm.cgmh.org.tw (C.-C.L.); lidesiao@livemail.tw (L.-D.H.); 2 Department of Anesthetics, College of Medicine, Chang Gung University, Kwei-San, Tao-Yuan 333, Taiwan; 3 Department of Physiology and Pharmacology and Health Aging Research Center, College of Medicine, Chang Gung University, Kwei-San, Tao-Yuan 333, Taiwan; royeariel760918@gmail.com; 4 Research Center for Chinese Herbal Medicine and Research Center for Food and Cosmetic Safety, College of Human Ecology, Chang Gung University of Science and Technology, Tao-Yuan 333, Taiwan

**Keywords:** CORM-2, heme oxygenase-1, NADPH oxidase, ROS, Nrf2, AREs

## Abstract

The upregulation of heme oxygenase-1 (HO-1) by the carbon monoxide-releasing molecule (CORM)-2 may be mediated through the activation of nicotinamide adenine dinucleotide phosphate (NADPH) oxidases [Nox] and reactive oxygen species (ROS) generation, which could provide cytoprotection against various cellular injuries. However, the detailed mechanisms of CORM-2-induced HO-1 expression in human pulmonary alveolar epithelial cells (HPAEpiCs) remain largely unknown. Therefore, we dissected the mechanisms underlying CORM-2-induced HO-1 expression in HPAEpiCs. We found that the administration of mice with CORM-2 attenuated the tumor necrosis factor-alpha (TNF-α)-induced intercellular adhesion molecule-1 (ICAM-1) expression and leukocyte count as revealed by immunohistochemical staining, western blot, real-time polymerase chain reaction (PCR), and cell count. Furthermore, TNF-α-induced ICAM-1 expression associated with monocyte adhesion to HPAEpiCs was attenuated by infection with adenovirus (adv)-HO-1 or incubation with CORM-2. These inhibitory effects of HO-1 were reversed by pretreatment with hemoglobin (Hb). Moreover, CORM-2-induced HO-1 expression was mediated via the phosphorylation of p47*^phox^*, c-Src, epidermal growth factor receptor (EGFR), Akt, and NF-E2-related factor 2 (Nrf2), which were inhibited by their pharmacological inhibitors, including diphenyleneiodonium (DPI) or apocynin (APO), ROS [N-acetyl-L-cysteine (NAC)], PP1, AG1478, PI3K (LY294002), or Akt (SH-5), and small interfering RNAs (siRNAs). CORM-2-enhanced Nrf2 expression, and anti-oxidant response element (ARE) promoter activity was also inhibited by these pharmacological inhibitors. The interaction between Nrf2 and AREs was confirmed with a chromatin immunoprecipitation (ChIP) assay. These findings suggest that CORM-2 increases the formation of the Nrf2 and AREs complex and binds with ARE-binding sites via Src, EGFR, and PI3K/Akt, which further induces HO-1 expression in HPAEpiCs. Thus, the HO-1/CO system might suppress TNF-α-mediated inflammatory responses and exert a potential therapeutic strategy in pulmonary diseases.

## 1. Introduction

Heme oxygenase (HO), a rate-limiting enzyme, metabolizes heme into biliverdin-IXα, ferrous iron, and carbon monoxide (CO). These secondary products are involved in the regulation of different physiological processes. Three isoforms of HO (HO-1, HO-2, and HO-3) have been characterized [1,2]. HO-1 is inducible and directly contributes to protect various organs from oxidative damage [1,3]. Moreover, CO has been shown to exert several biological functions, including anti-apoptotic and anti-inflammatory effects, which are mediated via the upregulation of HO-1 [4,5,6]. However, several reports have indicated that pro-inflammatory cytokines and oxidative stresses can also trigger HO-1 expression [7,8,9]. For example, HO-1 is induced by various factors in the airway cells of asthmatic patients [10]. Accumulating evidence concerning HO-1/CO-dependent cytoprotection elicits the mechanisms involved in the modulation of the inflammatory responses, including the downregulation of pro-inflammatory mediators, atherosclerosis, ischemia-reperfusion systems, and airway disorders [6,11,12]. Thus, the HO-1/CO system possesses beneficial effects via their potential against inflammatory, apoptotic, and proliferative events.

NADPH oxidase (Nox)-derived ROS generation has been approved to regulate either the expression of inflammatory or anti-inflammatory mediators in airway and pulmonary diseases [13]. Excessive ROS production, stimulated by several pro-inflammatory mediators, can regulate the expression of various inflammatory genes in airway disorders [13,14]. In contrast, low levels of ROS contribute to maintain cellular redox homeostasis and protect cells against oxidative stress under physiological conditions. Several studies have indicated that the exogenous application of CO and HO-1 could protect against oxidative stress and hyperoxic injury in the lung [15,16], gastric damage [17], and sepsis [6]. It has also been reported that the upregulation of the HO-1 protein via the Nox activity and intracellular ROS formation is induced by various stimuli such as lipopolysaccharides (LPS) and cytokines [6,18]. In addition, our previous reports demonstrated that the Nox/ROS system is a key player in HO-1 expression induced by lipotechoic acid (LTA) and cigarette smoke particle extract (CSPE) in human tracheal smooth muscle cells (HTSMCs) [19,20]. Moreover, our studies and others have demonstrated that the carbon monoxide-releasing molecule (CORM)-2 mediates Nox-dependent ROS generation in brain astrocytes [21,22] and human bronchial smooth muscle cells [23]. The Nox complex consists of two membrane-bound components, gp91*^phox^* and p22*^phox^*, and some cytosolic regulatory subunits, including p40*^phox^*, p47*^phox^*, p67*^phox^*, and the small GTPase Rac (Rac1 or Rac2). Upon enzyme activation, the cytoplasmic units translocate to the cell membrane, where they are assembled with gp91*^phox^*/p22*^phox^*, leading to ROS generation [19]. Thus, we applied CORM-2 as a potent inducer of the HO-1 gene and investigated the molecular mechanisms by which CORM-2 induced HO-1 gene expression in human pulmonary alveolar epithelial cells (HPAEpiCs).

CORMs have been extensively confirmed to provide an exogenous CO source and induce HO-1 expression in several cell types [6,7,24]. However, the roles of Nox/ROS involved in CORM-2-induced HO-1 expression are still unknown. HO-1 expression is regulated by various intracellular signaling pathways, such as ROS, growth factor receptors [(EGFR or platelet -derived growth factor receptor (PDGFR)], non-receptor tyrosine kinases (c-Src), or PI3K/Akt [25,26,27,28,29]. We also noticed that many transcription factors and stress-activated response elements in the upstream region of HO-1 promoter, such as Nrf2 and AREs are involved in the expression of HO-1 in response to oxidative stresses [30,31]. Upon exposure to oxidative stress, Nrf2 is dissociated from the cytoplasmic sequester Keap1 and translocated into the nucleus where it is exerted as an important transcription factor that regulates the expression of antioxidant genes by binding to AREs in the HO-1 promoter region [32]. Moreover, Nrf2 activation is mediated via various signaling pathways, such as PI3K/Akt [33], ROS [34], and c-Src [20]. Therefore, the roles of Nox/ROS, c-Src, EGFR, PI3K/Akt, and Nrf2/AREs in CORM-2-induced HO-1 expression were investigated in HPAEpiCs.

Furthermore, several reports have shown that when the exogenous application of the HO-1 end-product CO is administered at low concentrations, or alternatively, by pharmacological application of CORMs, it can also confer protective effects in models of inflammatory response and tissue injury [27,35,36]. Overexpression of HO-1 by cobalt protoporphyrin-9 (CoPPIX) can reduce TNF-α-induced oxidative stress and airway inflammation [5]. However, the detailed mechanisms by which CORM-2 induces HO-1 expression in HPAEpiCs are not completely understood. The experiments in this study were performed to dissect the mechanisms by which CORM-2 induces HO-1 expression mediated via Nrf2/AREs activation in HPAEpiCs and suppressed TNF-α-mediated inflammatory responses. These findings suggested that in HPAEpiCs, CORM-2-induced HO-1 expression is, at least in part, mediated through a Nox2/ROS/c-Src/EGFR/ PI3K/Akt-dependent Nrf2/AREs pathway.

## 2. Experimental Section

### 2.1. Reagents and Antibodies

Dulbecco’s modified eagle medium (DMEM)/F-12 medium, fetal bovine serum (FBS), TRIzol reagent, and PLUS-Lipofectamine were from Invitrogen (Carlsbad, San Diego, CA, USA). PP1, AG1478, apocynin (APO), diphenyleneiodonium chloride (DPI), N-acetyl cysteine (NAC), LY294002, and SH-5 were from Biomol (Plymouth Meeting, Montgomery, PA, USA). The luciferase assay kit was from Promega (Madison, Dane, WI, USA). Anti-HO-1 (Cat# ADI-SPA-895), anti-ICAM-1 (H-108) (sc-7891), anti-β-actin (sc-47778), anti-EGFR (sc-373746), anti-c-Src (sc-18), anti-Nox2 (ab129068), anti-p47*^phox^* (sc-14015), anti-p110 (sc-7189), anti-Nrf2 (sc-722), and anti-lamin A (sc-20680) antibodies were from Santa Cruz Biotechnology (Dallas, TX, USA). Anti-phospho-c-Src (#2101), anti-phospho-EGFR (#2231), and anti-phospho-Akt (#9271) antibodies were from Cell Signaling (Danvers, Essex, MA, USA). Anti-phospho-Nrf2 antibody (ab76026) was from Abcam (Cambridge, Cambridgeshire, UK). Tricarbonyldichlororuthenium (II) dimer (CORM-2) and other chemicals were from Sigma-Aldrich (St. Louis, MO, USA). 5-(and-6)-chloromethyl-2′,7′-dichlorodihydrofluorescein diacetate, acetyl ester (CM-H_2_DCFDA) were from Molecular Probes (Eugene, Lane, OR, USA). SDS-PAGE reagents were from MDBio Inc (Taipei, Taiwan).

### 2.2. Animal Care and Experimental Procedures

Male institute of cancer research (ICR) mice aged 6–8 weeks were purchased from the National Laboratory Animal Centre (Taipei, Taiwan). The experimental procedures were conducted according to the animal ethics committee of the Animal Care Committee of Chang Gung University (Approval Document No. CGU 16-046) and the National Institutes of Health (NIH) Guides for the Care and Use of Laboratory Animals. Animals were assigned randomly to different experimental groups for all in vivo studies. Data collection and evaluation of all in vivo and in vitro experiments were performed blindly with respect to group identity. Mice were divided into 5 groups with 5 mice in each group/cage and kept in standard, individually ventilated cages in an animal facility under standardized conditions (12 h light/dark cycle, 21–24 °C, humidity of 50–60%) with food and water ad libitum. For the in vivo treatment, zinc protoporphyrin-9 (ZnPPIX) and CORM-2 were dissolved in dimethyl sulfoxide (DMSO) at a concentration of 10 and 50 mg/mL, respectively, and further diluted in phosphate buffered saline (PBS) before injection. The final dosages for ZnPPIX and CORM-2 were 2 and 10 mg/kg intraperitoneal (i.p.), respectively. The control mice were given 0.1 mL of DMSO-PBS with 0.1% bovine serum albumin (BSA). Mice were anesthetized by i.p. injection of 200 μL pentobarbital sodium (5 mg/mL). The depth of anesthesia was evaluated by pinching the animal’s paw with forceps, and all efforts were made to minimize suffering. Mice were placed individually on a board in a near-vertical position, and their tongues were withdrawn with lined forceps. Mice were i.p. given one dose of ZnPP IX (2 mg/kg) for 2 h or CORM-2 (10 mg/kg) for 24 h. TNF-α (0.75 mg/kg body weight) was placed posterior in the throat and aspirated into the lungs. After 24 h, the mice were sacrificed under isoflurane anesthesia, and then, specimens were harvested. Bronchoalveolar lavage (BAL) fluid was performed through a tracheal cannula using 1 mL aliquots of ice-cold PBS solution. BAL fluid was centrifuged at 500× *g* at 4 °C, and cell pellets were washed and re-suspended in PBS. Leukocyte count was determined by a hemocytometer, as previously described [5]. To examine the levels of HO-1 and ICAM-1 expression in the mice treated with or without CORM-2 followed by treatment with TNF-α, lung tissues were collected, homogenized, and subjected to western blot analysis to determine the levels of ICAM-1, HO-1, and β-actin protein, as previously described [5].

### 2.3. Cell Culture and Treatment

Human pulmonary alveolar epithelial cells (HPAEpiCs) were purchased from the ScienCell Research Laboratories (San Diego, CA, USA) and grown as previously described [37]. Passages of HPAEpiCs from 4 to 6 were used throughout this study.

### 2.4. Transient Transfection with siRNAs

Human siRNAs of scrambled, c-Src (SASI_Hs01_00112905), EGFR (SASI_Hs01_00215449), p47*^phox^* (SASI_Hs02_00302212), Nox2 (SASI_Hs01_00086110), p110 (SASI_Hs01_00219338), and Nrf2 (SASI_Hs02_00302212) were from Sigma (St. Louis, MO, USA). Transient transfection of siRNAs (100 nM) was performed using a GeneMute reagent according to the manufacturer’s instructions from SignaGen Laboratories (Rockville, Montgomery, MD, USA).

### 2.5. Real-Time RT-PCR

Total RNA was extracted from HPAEpiC using TRIzol reagent. RNA concentration was spectrophotometrically determined at 260 nm. mRNA was reverse-transcribed into cDNA and analyzed by real-time quantitative PCR (RT-qPCR). RT-qPCR was performed with a 7500 Real-Time PCR System (Applied Biosystems, Foster City, San Mateo, CA, USA) and KAPA PROBE FAST ABI Prism^®^ qPCR kit (KK4705, Kapa Biosystems, Wilmington, MA, USA) using primers and probe mixes for HO-1, ICAM-1, and endogenous glyceraldehyde 3-phosphate dehydrogenase (GAPDH) control genes. The expression of HO-1 and ICAM-1 were quantified by normalization to the GAPDH expression. Relative gene expression was determined by the ΔΔ^Ct^ method, where Ct is the threshold cycle. All the experiments were performed in triplicate (*n* = 3).

### 2.6. Preparation of Cell Extracts and Western Blot

Growth-arrested HPAEpiCs were incubated with CORM-2 at 37 °C for the indicated time intervals. The cells were washed, scraped, collected, and centrifuged at 45000× *g* at 4 °C for 1 h to yield the whole cell extract, as previously described [19]. Samples were denatured, subjected to SDS-PAGE using a 12% running gel, and transferred to nitrocellulose membrane. Membranes were probed overnight with respective antibodies. Membranes were washed with Trinidad and Tobago Bureau of Standards (TTBS) 4 times for 5 min each and incubated with anti-rabbit or anti-mouse horseradish peroxidase antibody (1:2000) for 1 h. Follow incubation, the membranes were extensively washed with TTBS. The immunoreactive bands were detected by Enhanced chemiluminescence (ECL) reagents and captured with a UVP BioSpectrum 500 Imaging System (Upland, San Bernardino, CA, USA). The image densitometry analysis was quantified by an UN-SCAN-IT gel software (Orem, Utah, UT, USA).

### 2.7. Isolation of Subcellular Fractions

Cells were harvested and sonicated for 5 s at an output of 1.5 with a sonicator (Misonix, Farmingdale, Nassau, NY, USA), and centrifuged at 4700× *g* for 15 min at 4 °C. The pellet was collected as the nuclear fraction. The supernatant was centrifuged at 14000× *g* at 4 °C for 60 min to yield the pellet (membrane fraction) and the supernatant (cytosolic fraction).

### 2.8. Immunofluorescence Staining

Growth-arrested HPAEpiCs were incubated with CORM-2 for the indicated time intervals. After washing twice with ice-cold PBS, cells were fixed, permeabilized, and stained using an anti-Nrf2 antibody as previously described [19]. The images were observed using a fluorescence microscope (Zeiss, Axiovert 200M, Oberkochen, Baden-Württemberg, Germany).

### 2.9. Chromatin Immunoprecipitation (ChIP) Assay

To detect the association of nuclear proteins with human HO-1 promoter, ChIP analysis was conducted as previously described [38]. DNA immunoprecipitated by an anti-Nrf2 antibody was purified. The DNA pellet was re-suspended in H_2_O and subjected to PCR amplification with the forward primer 5′-TCCTTTCCCGAGCCACGTG-3′ and the reverse primer 5′-TCCGGACTTTGCCCCAGG-3′, which were specifically designed from the HO-1 promoter ARE region (−9107 to −8909). PCR products were analyzed on ethidium bromide-stained agarose gels (2%).

### 2.10. Co-Immunoprecipitation Assay

Cell lysates containing 1 mg of protein were incubated with 2 µg of an anti-EGFR or anti-Nox2 antibody at 4 °C for 24 h, then 10 µL of 50% protein A-agarose beads was added and mixed at 4 °C for 24 h. The immunoprecipitates were collected and washed thrice with a lysis buffer without Triton X-100. 5X Laemmli buffer was added and subjected to electrophoresis on 10% SDS-PAGE and then blotted using an anti-c-Src, anti-EGFR, anti-p47*^phox^*, or anti-Nox2 antibody.

### 2.11. ARE Promoter Activity

To obtain the ARE-luciferase reporter construct, double-stranded oligonucleotides containing a single copy of the 41-bp pair murine GST-Ya ARE (5′-TAGCTTGGAAATGACATTGCTAATGGTGACAAAGCAACTTT-3′; the core sequence underlined) were cloned into the pGL2 promoter vector (Promega, Madison, Dane, WI, USA). All sequences of pARE-Luci were confirmed and verified the presence of the correct sequence and the absence of any other nucleotide changes by DNA sequencing. ARE-Luci activity was determined as previously described using a luciferase assay system (Promega, Madison, Dane, WI, USA) [19].

### 2.12. Statistical Analysis of Data

All data were expressed as the mean or mean ± S.E.M of three individual experiments performed in duplicate or triplicate. The significance of difference between two groups was determined by a paired two-tailed Student’s *t*-test for western blot data. All other statistical analyses were a comparison of multiple groups; a GraphPad Prism Program (GraphPad, San Diego, CA, USA) by an unpaired *t*-test or one-way analysis of variance (ANOVA), followed by Tukey’s post-hoc test, was used. A *p* < 0.05 value was considered significant.

## 3. Results

### 3.1. CORM-2 Inhibits TNF-α-Induced Lung Inflammation in Mice

**Figure 1:**
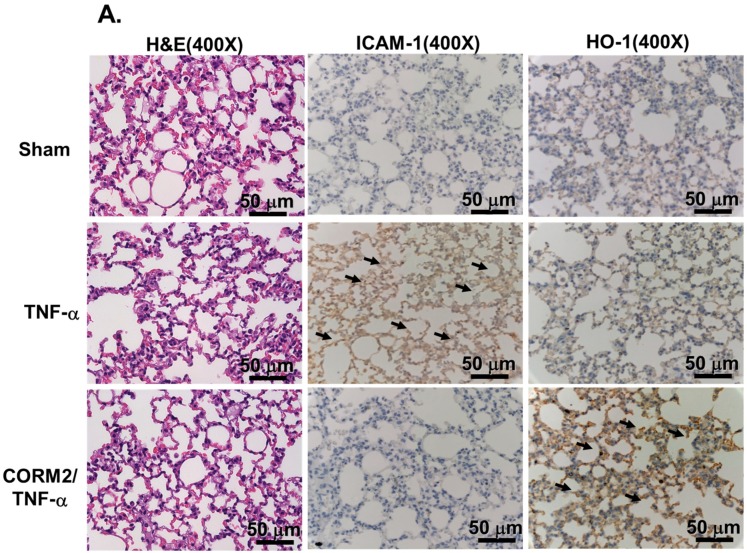

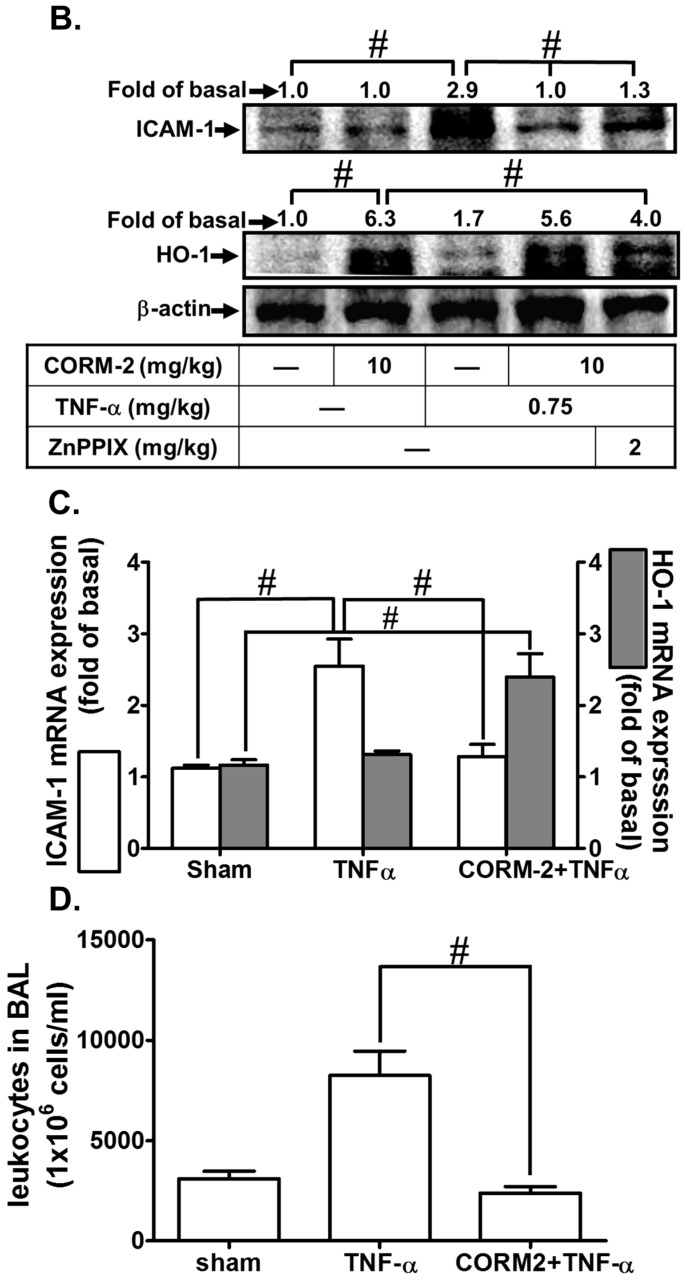
CORM-2 attenuated TNF-α-induced pulmonary inflammatory responses in vivo. Mice were intra-peritoneal pretreated with CORM-2 (10 mg/kg) or a vehicle for 1 h and then intra-tracheally administered with or without TNF-α (0.75 mg/kg) for 16 h. (**A**) Hematoxylin/Eosin (H/E) and immunohistochemical staining for ICAM-1 and HO-1 in serial sections of lung tissues from Sham (PBS-treated mice), TNF-α-treated mice, and CORM-2 + TNF-α-treated mice. The arrow indicates pulmonary alveolar cells displayed with ICAM-1 and HO-1 expression. (**B**,**C**) Lung tissues were homogenized to extract protein and mRNA. The protein and mRNA levels of ICAM-1 and HO-1 were determined by western blot and mRNA. (**D**) BAL fluid was acquired, and leukocyte count was determined by a hemocytometer. Data are expressed as mean ± SEM, *n* = 5. ^#^ *p* < 0.01, as compared between the indicated groups.

In our previous studies, TNF-α has been shown to induce the expression of vascular cell adhesion protein-1 (VCAM-1) or ICAM-1 in various cell types [12,39]. First, we investigated the anti-inflammatory effects of CORM-2 on TNF-α-induced lung inflammation. In an animal study, TNF-α markedly induced ICAM-1 protein and mRNA expression in the lung tissues and the leukocyte count in BAL fluid, which were also reduced by pretreatment with CORM-2 (Figure 1). These inhibitory effects of CORM-2 were mediated via the upregulation of HO-1, which were reversed by pretreatment with the inhibitor of HO-1 activity (ZnPP IX) (Figure 1B), suggesting that HO-1 induction by CORM-2 protects against inflammatory responses in mice challenged with TNF-α.

### 3.2. Adenovirus-Mediated HO-1 Expression Attenuates TNF-α-Induced Inflammatory Responses In Vitro

**Figure 2:**
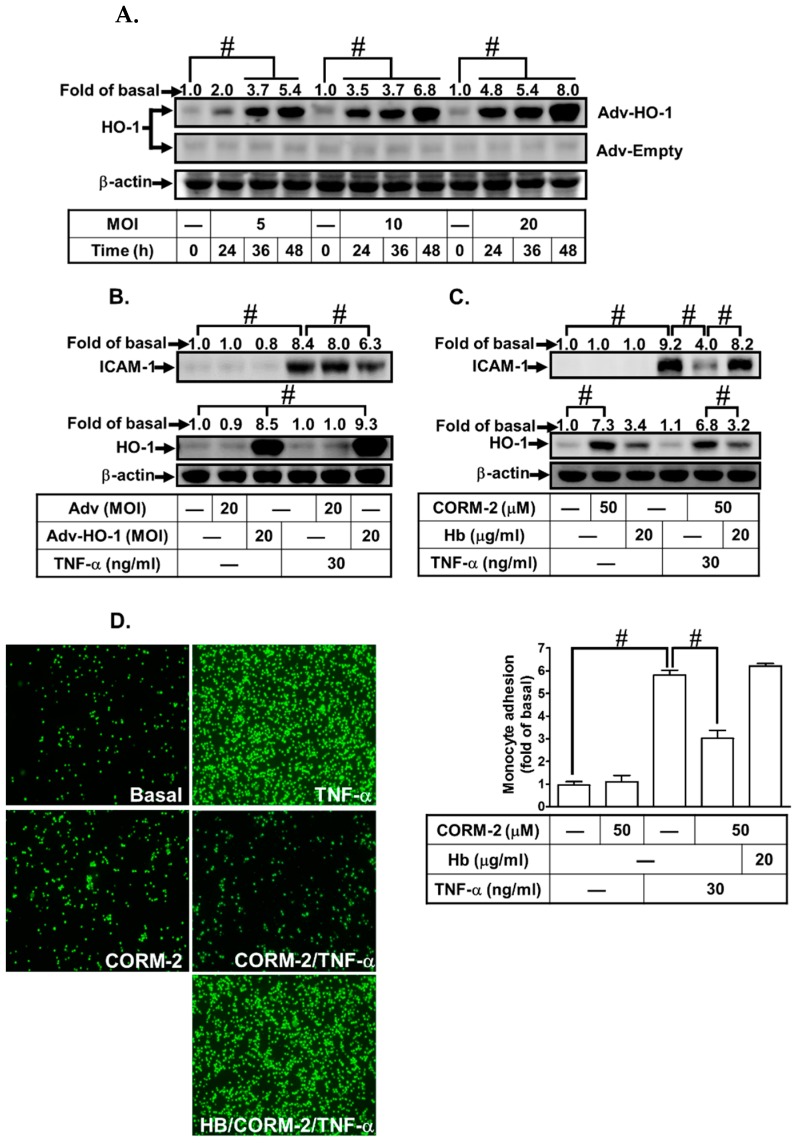
Upregulation of HO-1 suppresses TNF-α-induced ICAM-1 expression on human pulmonary alveolar epithelial cells (HPAEpiCs). (**A**) Cells were infected with an indicated multiplicity of infection (MOI) with either an empty adenovirus (Adv) or Adv-HO-1 for the indicated time intervals. (**B**) HPAEpiCs were infected with 20 MOI of empty Adv or Adv-HO-1 for 36 h, followed by 30 ng/mL TNF-α for 10 h. (**C**,**D**) HPAEpiCs were pretreated with 20 μg/mL hemoglobin (Hb) for 1 h and then treated with 50 μM CORM-2 for 6 h, followed by incubation with 30 ng/mL TNF-α for 10 h. (**A**–**C**) The levels of HO-1, ICAM-1, and β-actin protein were determined by western blot. (**D**) The adhesion of THP-1 cells was measured. Data are expressed as mean ± SEM, *n* = 3. ^#^ *p* < 0.01, as compared with the respective groups as indicated.

To ensure the effect of HO-1 expression on TNF-α-induced responses, HPAEpiCs were infected with an empty Adv or Adv-HO-1 for the indicated time periods. Infection of HPAEpiCs with Adv-HO-1, but not Adv alone significantly induced HO-1 expression in a time- and concentration-dependent manner (Figure 2A). TNF-α-induced ICAM-1 expression and monocyte adherence was attenuated in HPAEpiCs infected with Adv-HO-1 or pretreatment with CORM-2, which was significantly reversed by hemoglobin (Hb) (Figure 2B–D). These results confirmed that upregulation of HO-1 by CORM-2 or Adv-HO-1 protects against the inflammatory responses in HPAEpiCs exposed to TNF-α.

### 3.3. CORM-2 Stimulates Nox2/ROS-Dependent HO-1 Expression

**Figure 3:**
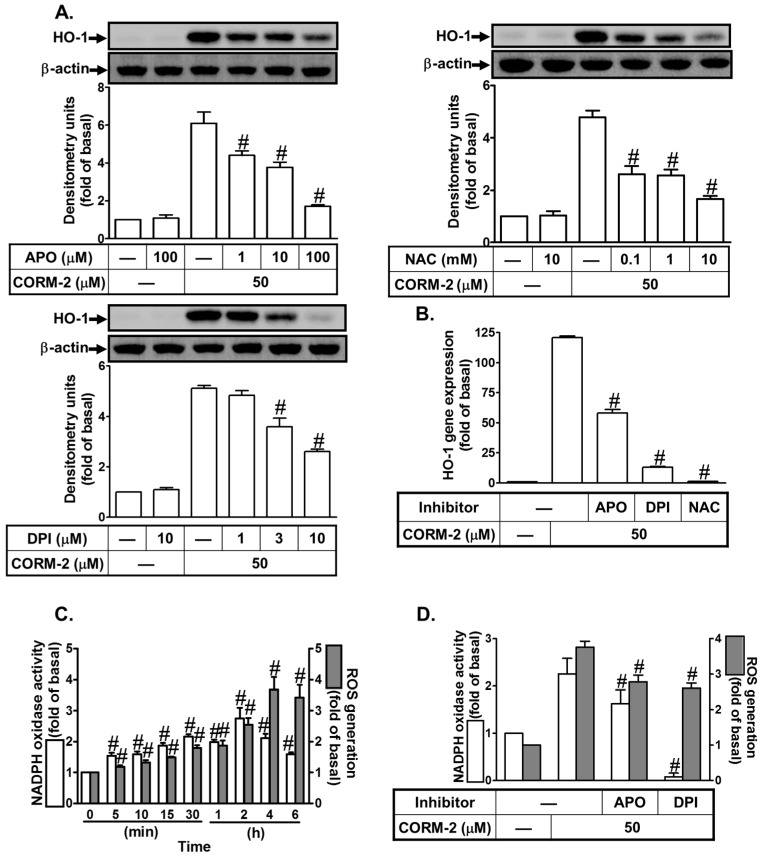
CORM-2 induces HO-1 expression via Nox/ROS. (**A**) HPAEpiCs were pretreated with APO, DPI, or NAC for 2 h and then incubated with CORM-2 for 16 h. The protein levels of HO-1 and β-actin were determined by western blot. (**B**) Cells were pretreated with APO (100 μM), DPI (10 μM), or NAC (10 mM) for 2 h and then incubated with CORM-2 for 6 h. The mRNA expression of HO-1 was determined by real-time PCR. (**C**) Cells were treated with CORM-2 for the indicated time intervals. Nox activity and ROS generation were measured. (**D**) Cells were pretreated with APO (100 μM) or DPI (10 μM) and then incubated with CORM-2 for 2 h or 4 h. Nox activity and ROS generation were measured. Data are expressed as mean ± S.E.M. of three independent experiments (*n* = 3). ^#^ *p* < 0.01, as compared with the cells exposed to the vehicle alone (**C**) or CORM-2 alone (**A**,**B**,**D**).

Excessive production of ROS by Nox is thought to be responsible for tissue injury associated with a range of respiratory inflammatory diseases [13,14]. Nox/ROS has been shown to be involved in HO-1 induction in various cell types [19,40]. In this study, we found that pretreatment with the inhibitor of Nox (DPI or APO) or ROS (NAC) markedly reduced CORM-2-induced HO-1 protein and mRNA levels (Figure 3A,B). On the other hand, we showed that in HPAEpiCs, CORM-2 time-dependently induced intracellular Nox activation and ROS generation (Figure 3C). We previously indicated that c-Src and EGFR played key roles in mediating Nox activation and ROS production [13]. In this study, we also showed that CORM-2-induced Nox activation and ROS generation was reduced by APO or DPI (Figure 3D). These results suggest that CORM-2-induced HO-1 expression is mediated through Nox/ROS generation in HPAEpiCs.

**Figure 4:**
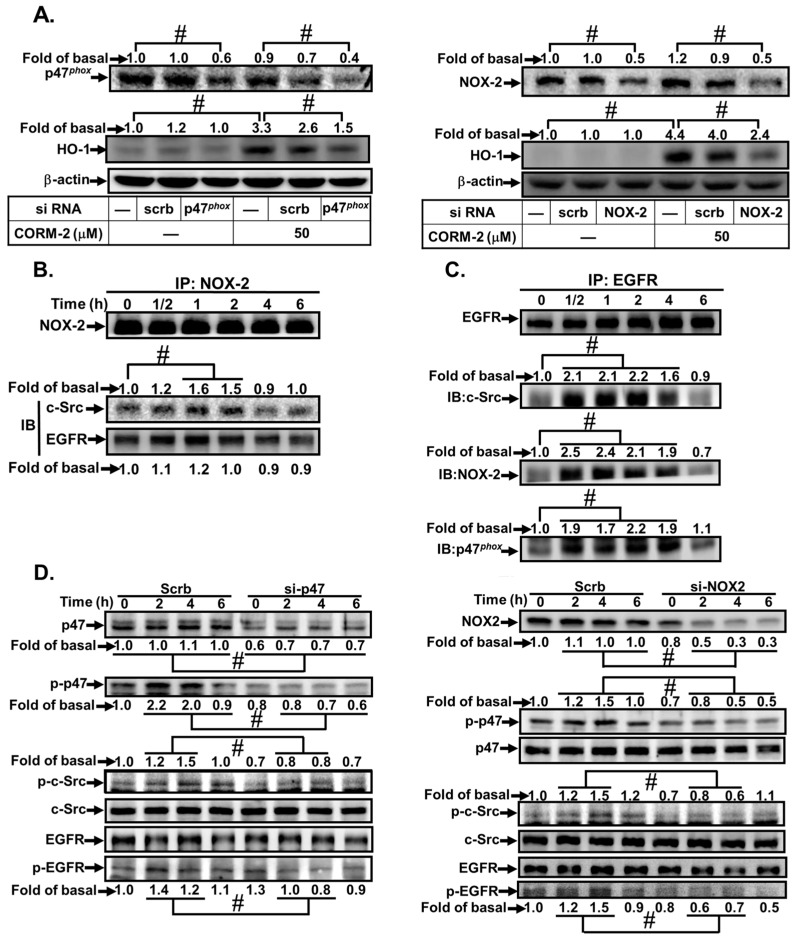
CORM-2 induces the formation of a c-Src/EGFR/p47*^phox^*/Nox2 complex. (**A**) HPAEpiCs were transfected with scrambled, p47*^phox^*, or Nox2 siRNA and then incubated with CORM-2 for 16 h. The levels of p47*^phox^*, Nox2, HO-1, and β-actin protein were determined by western blot. (**B**,**C**) Cells were treated with CORM-2 for the indicated time intervals. The cell lysates were subjected to immunoprecipitation using an anti-Nox2 or anti-EGFR antibody. The immunoprecipitates were analyzed by western blot using an anti-c-Src, anti-EGFR, anti-Nox2, or anti-p47*^phox^* antibody. (**D**) HPAEpiCs were transfected with scrambled, p47*^phox^*, or Nox2 siRNA and then incubated with CORM-2 for the indicated time intervals. The levels of p47*^phox^*, c-Src, and EGFR phosphorylation were determined by western blot. Data are representative of three independent experiments (*n* = 3). ^#^ *p* < 0.01, as compared with the respective values of cells stimulated with CORM2 alone or compared between the indicated groups.

The p47*^phox^* regulatory subunit plays a critical role in the activation of Nox; phosphorylation of p47*^phox^* is thought to relieve inhibitory intracellular interactions and permit the binding of p47*^phox^* to p22*^phox^*, thereby increasing oxidase activation [19]. To further investigate the roles of Nox2 and p47*^phox^* in CORM-2-induced HO-1 expression, as shown in Figure 4A, transfection with either Nox2 or p47*^phox^* siRNA knocked down the level of Nox2 or p47*^phox^* protein and markedly inhibited CORM-2-induced HO-1 expression. We previously demonstrated that lipoteichoic acid induces the formation of a c-Src/p47*^phox^* complex leading to HO-1 expression [19]. Here, we investigated the relationship of c-Src, EGFR, p47*^phox^*, and Nox2 in CORM-2-challenged HPAEpiCs. As shown in Figure 4B,C, cells were stimulated with CORM-2 for the indicated time periods. The cell lysates were subjected to immunoprecipitation using an anti-Nox2 or anti-EGFR antibody, and then, the immunoprecipitates were analyzed by western blot using an anti-c-Src, anti-EGFR, anti-Nox2, or anti-p47*^phox^* antibody. The protein levels of c-Src and EGFR were time-dependently increased in a Nox2-immunoprecipitated complex. Similarly, cell lysates immunoprecipitated using an EGFR antibody also enhanced the association among c-Src, p47*^phox^*, and Nox2. Furthermore, the relationship between p47*^phox^*/Nox2 and c-Src/EGFR phosphorylation was differentiated by transfection with either p47*^phox^* or Nox2 siRNA, which knocked down the level of p47*^phox^* or Nox2 protein and attenuated p47*^phox^*, c-Src, and EGFR phosphorylation stimulated by CORM-2 (Figure 4D). These results suggest that CORM-2 triggers the interaction among p47*^phox^*/Nox2-dependent ROS generation, leading to c-Src and EGFR activation to induce HO-1 expression in HPAEpiCs.

### 3.4. CORM-2 Induces HO-1 Expression via a c-Src/EGFR Pathway

**Figure 5:**
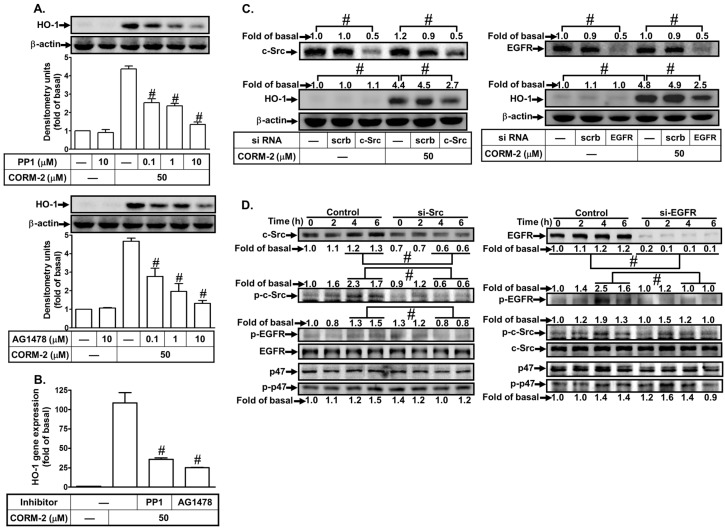
CORM-2 induces c-Src/EGFR-dependent HO-1 expression. (**A**) HPAEpiCs were pretreated with PP1 or AG1478 for 1 h and then incubated with CORM-2 for 16 h. The protein levels of HO-1 and β-actin were determined by western blot. (**B**) Cells were pretreated with PP1 (10 μM) or AG1478 (10 μM) for 1 h and then incubated with CORM-2 for 6 h. The mRNA expression of HO-1 was determined by real-time PCR. (**C**) Cells were transfected with scrambled, c-Src, or EGFR siRNA and then incubated with CORM-2 for 16 h. The levels of c-Src, EGFR, HO-1, and β-actin protein were determined by western blot. (**D**) Cells were transfected with scrambled, c-Src, or EGFR siRNA and then incubated with CORM-2 for the indicated time intervals. The levels of c-Src, EGFR, p47, phospho-c-Src, phospho-EGFR, phospho-p47, and β-actin protein were determined by western blot. Data are expressed as mean ± S.E.M. of three independent experiments (*n* = 3). ^#^ *p* < 0.01, as compared with the cells exposed to CORM-2 alone or compared between the indicated groups.

Previous studies have shown that c-Src is involved in EGFR transactivation [41,42] leading to HO-1 induction [13,26]. Therefore, the roles of c-Src and EGFR in CORM-2-induced HO-1 expression were investigated in HPAEpiCs. We found that pretreatment with an inhibitor of either c-Src (PP1) or EGFR (AG1478) markedly inhibited CORM-2-induced HO-1 protein levels (Figure 5A) and mRNA expression (Figure 5B). We further confirmed the roles of c-Src and EGFR in CORM-2-induced HO-1 expression by transfection with either c-Src or EGFR siRNA, which knocked down the level of c-Src or EGFR protein and then inhibited the CORM-2-induced HO-1 protein expression (Figure 5C). Whether HO-1 expression was mediated through c-Src and EGFR phosphorylation was determined by Western blot. As shown in Figure 5D, transfection with c-Src siRNA attenuated c-Src and EGFR phosphorylation but not p47*^phox^* phosphorylation stimulated by CORM-2, while transfection with EGFR siRNA reduced CORM-2-stimulated EGFR phosphorylation but had no effect on p47*^phox^* and c-Src phosphorylation. These results suggest that CORM-2-induced HO-1 expression is mediated through c-Src/EGFR in HPAEpiCs.

### 3.5. CORM-2 Stimulates PI3K/Akt-Dependent HO-1 Expression

**Figure 6:**
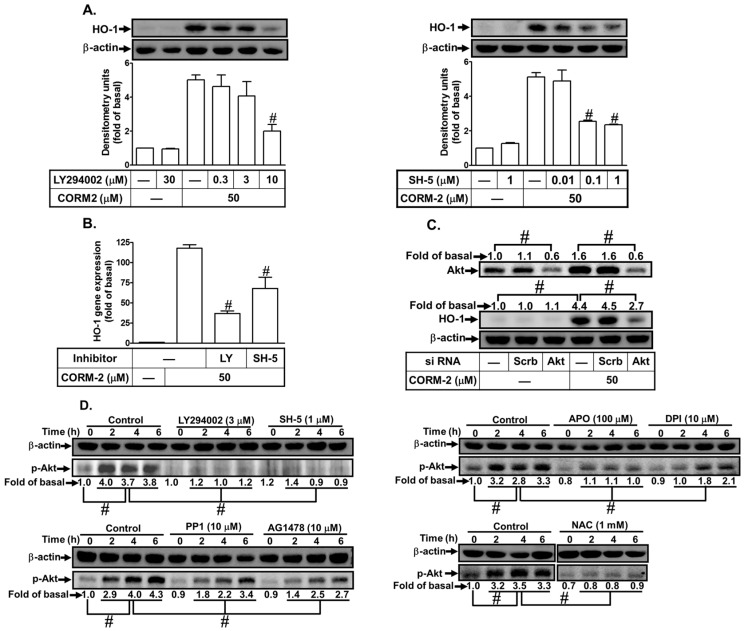
CORM-2 induces HO-1 expression via PI3K/Akt. (**A**) HPAEpiCs were pretreated with LY294002 or SH-5 for 1 h and then incubated with CORM-2 for 16 h. The protein levels of HO-1 and β-actin were determined by western blot. (**B**) Cells were pretreated with LY294002 (10 μM) or SH-5 (1 μM) for 1 h and then incubated with CORM-2 for 6 h. The mRNA expression of HO-1 was determined by real-time PCR. (**C**) Cells were transfected with scrambled or Akt siRNA and then incubated with CORM-2 for 16 h. The levels of Akt, HO-1, and β-actin protein were determined by western blot. (**D**) Cells were pretreated without or with LY294002, SH-5, PP1, AG1478, APO, DPI, or NAC for 1 h and then incubated with CORM-2 for the indicated time intervals. The protein levels of phospho-Akt and β-actin were determined. Data are expressed as mean ± S.E.M. of three independent experiments (*n* = 3). ^#^ *p* < 0.01, as compared with the cells exposed to CORM-2 alone or compared between the indicated groups.

PI3K/Akt has been shown to be involved in HO-1 induction [29]. Therefore, the roles of PI3K/Akt in CORM-2-induced HO-1 expression were investigated in HPAEpiCs. We found that pretreatment with the inhibitor of PI3K (LY294002) or Akt (SH-5) markedly inhibited CORM-2-induced HO-1 protein levels (Figure 6A) and mRNA expression (Figure 6B). To confirm the role of PI3K/Akt in CORM-2-induced HO-1 expression, as shown in Figure 6C, transfection with Akt siRNA knocked down the level of Akt protein and then inhibited CORM-2-induced HO-1 protein expression. In addition, we investigated whether HO-1 expression was mediated through Akt phosphorylation. As shown in Figure 6D, CORM-2-induced Akt phosphorylation was inhibited by LY294002, SH-5, PP1, AG1478, APO, DPI, or NAC. These results suggest that CORM-2-induced HO-1 expression is mediated through a Nox/ROS/c-Src/EGFR-dependent Akt pathway in HPAEpiCs.

### 3.6. CORM-2 Stimulates Nrf2/ARE-Dependent HO-1 Expression

**Figure 7:**
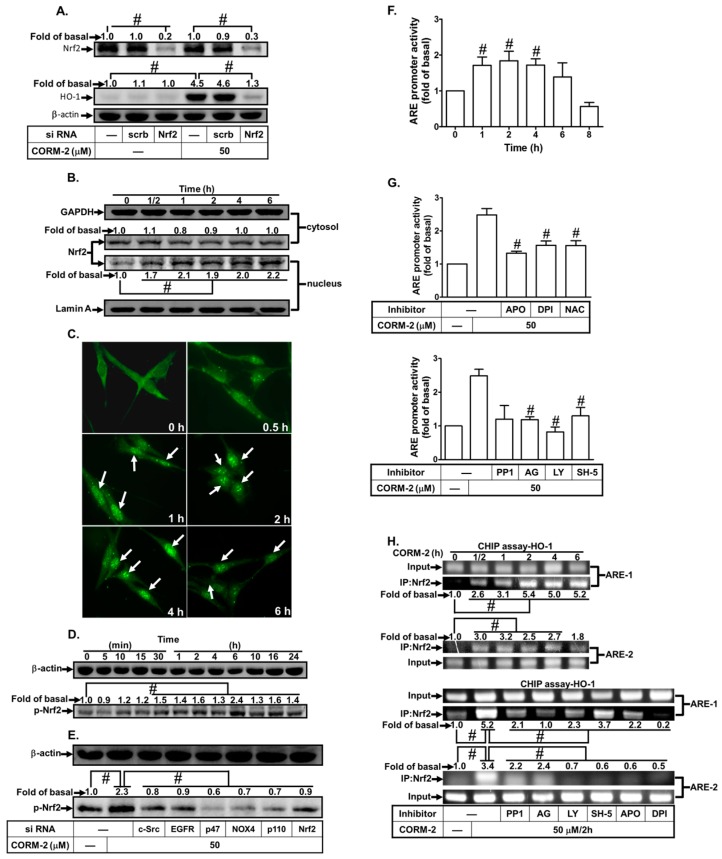
CORM-2 induces HO-1 expression via Nrf2. (**A**) HPAEpiCs were transfected with scrambled or Nrf2 siRNA and then incubated with CORM-2 for 16 h. The levels of Nrf2, HO-1, and β-actin protein were determined by western blot. (**B**) Cells were treated with CORM-2 for the indicated time intervals. The cytosol and nucleus fractions were prepared and subjected to western blot using an anti-Nrf2 antibody. GAPDH and lamin A were used as marker proteins for cytosol and nucleus fractions, respectively. (**C**) Cells were treated with CORM-2 for the indicated time intervals. Cells were fixed, and then labeled with the anti-Nrf2 antibody and FITC-conjugated secondary antibody. Individual cells were imaged as described in Section 2. (**D**,**E**) Cells were treated with CORM-2 for the indicated time intervals or transfected with siRNA of c-Src, EGFR, p47*^phox^*, Nox2, or p110, and then incubated with CORM-2 for 6 h. The protein levels of phospho-Nrf2 and β-actin were determined. (**F**,**G**) ARE-luc plasmids transfected HPAEpiCs were pretreated (**F**) without or (**G**) with PP1, AG1478, LY294002, SH-5, APO, DPI, or NAC for 2 h and then incubated with vehicle or CORM-2 (30 μM) for 2 h. ARE promoter luciferase activity was determined in the cell lysates. (**H**) Cells were treated with CORM-2 for the indicated times or pretreated with PP1, AG1478, LY294002, SH-5, APO, or DPI, and then incubated with CORM-2 for 6 h. Nrf2 binding activities were analyzed by a ChIP assay. Data are expressed as mean ± S.E.M. of three independent experiments (*n* = 3). ^#^ *p* < 0.01, as compared with the cells exposed to the CORM-2 alone or compared between the indicated groups.

Nrf2 is an important transcription factor that regulates the expression of antioxidant genes through binding to AREs in the promoter region of HO-1 in response to ROS [19,43]. Therefore, the role of Nrf2 in CORM-2-induced HO-1 expression was investigated in HPAEpiCs. We found that transfection with Nrf2 siRNA knocked down the level of Nrf2 and markedly inhibited CORM-2-induced HO-1 protein expression (Figure 7A). CORM-2 also induced Nrf2 translocation from the cytosol to the nucleus, as determined by cell fractions coupled with western blot or immunofluorescence staining (Figure 7B,C). CORM-2 also time-dependently stimulated Nrf2 phosphorylation in HPAEpiCs (Figure 7D). Moreover, we investigated the relationship of Nox2, p47*^phox^*, c-Src, EGFR, PI3K, and Nrf2 in CORM-2-challenged HPAEpiCs. As shown in Figure 7E, transfection with Nox2, p47*^phox^*, c-Src, EGFR, or p110 siRNA markedly reduced CORM-2-stimulated Nrf2 phosphorylation. CORM-2 also time-dependently induced ARE promoter activity, which was inhibited by PP1, AG1478, LY294002, SH-5, APO, DPI, or NAC (Figure 7F,G). Finally, we used a ChIP assay to determine whether CORM-2-stimulated recruitment of Nrf2 to the HO-1 promoter was involved in HO-1 gene expression. Chromatin was immunoprecipitated by using an anti-Nrf2 antibody, and the ARE region (−9107 to −8909) of the HO-1 promoter was amplified by RT-PCR. As shown in Figure 7H, CORM-2 stimulated the binding of Nrf2 to ARE on the HO-1 promoter in a time-dependent manner, which was reduced by PP1, AG1478, LY294002, SH-5, APO, or DPI. These results suggest that CORM-2-induced HO-1 expression is mediated through a Nrf2-dependent ARE signaling pathway in HPAEpiCs.

## 4. Discussion

**Figure 8:**
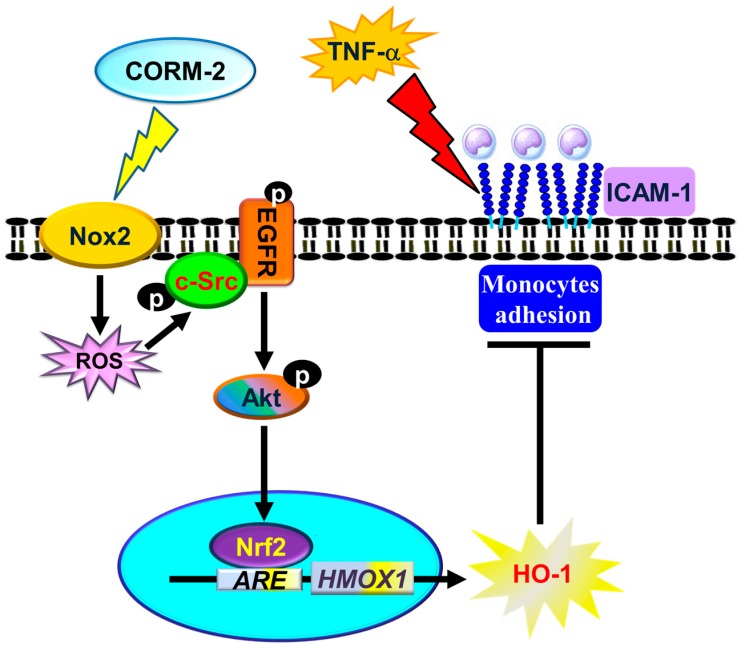
A model for the molecular mechanisms underlying CORM-2-induced HO-1 expression and activity in HPAEpiCs. CORM-2-induced HO-1 expression was mediated through Nox/ROS and c-Src/EGFR-dependent activation of PI3K/Akt, linking to the upregulation of Nrf2/AREs, which promoted HO-1 expression and enzymatic activity in HPAEpiCs. Upregulation of HO-1 suppressed the inflammatory responses triggered by TNF-α in both in vitro and in vivo studies.

The anti-inflammatory properties of CORMs have been demonstrated in several animal models, suggesting a possible therapeutic application for the treatment of inflammatory diseases. However, the roles of Nox/ROS in CORM-2-induced HO-1 expression have not been fully defined. Here, we observed that pretreatment with CORM-2 inhibited TNF-α-induced lung inflammatory responses via HO-1 induction. Furthermore, the application of selective pharmacological inhibitors and genetic silencing through transfection with siRNA of p47*^phox^*, Nox2, c-Src, EGFR, p110, Akt, or Nrf2 attenuated the CORM-2-induced HO-1 expression. The present study demonstrated that CORM-2-induced HO-1 expression is mediated through a Nox2/ROS/c-Src/EGFR/PI3K/Akt-dependent Nrf2/AREs pathway and suppresses the inflammatory responses triggered by TNF-α in both in vitro and in vivo studies (Figure 8).

ROS act as a messenger in normal physiological functions, and inflammatory responses are dependent on their cellular concentrations [44,45]. It has been reported that the upregulation of HO-1 protein due to Nox activity and intracellular ROS formation is induced by various stimuli, such as LPS or cytokines [6,18]. Our previous studies and others indicated that CORMs mediate the Nox-dependent ROS generation in brain astrocytes [21,22]. Therefore, Nox-dependent ROS generation is, at least in part, involved in the upregulation of HO-1 expression by CORM-2 in HPAEpiCs. We further differentiated the relationship between Nox-dependent ROS generation and HO-1 expression in HPAEpiCs challenged with CORM-2, and a thiol-containing compound (NAC) was used to scavenge ROS. NAC has been shown to reduce the injurious effects of hydrogen peroxide in human alveolar and bronchial epithelial cells [46]. Moreover, two Nox-related inhibitors, DPI (a Nox inhibitor) and APO (a p47*^phox^* inhibitor), have been shown to prevent p47*^phox^* (a Nox subunit) translocation to the plasma membrane and then inhibit Nox activation [47]. Our results showed that CORM-2-induced ROS generation and HO-1 expression were inhibited by DPI or APO, suggesting that Nox plays an important role in these responses. These data are consistent with previous reports showing that Nox-derived ROS generation is involved in HO-1 induction in HTSMCs [19,20]. Moreover, NAC is a widely used and powerful scavenger of hydrogen peroxide, hydroxyl radicals, superoxide, and hypochlorous acid in several models. Thus, pretreatment with NAC could scavenge the ROS generated by CORM-2 and then attenuate HO-1 expression in HPAEpiCs. Indeed, low levels of ROS can regulate cellular processes, such as proliferation, gene expression, immunity, and wound healing [48]. Conversely, higher levels of ROS can exert an antibacterial effect and cause cell damage and death [23,49]. Previous reports, as well as ours, also indicate that CO acts as a secondary messenger to mediate metabolism and gene expression including HO-1 [50,51]. These results strongly support the involvement of Nox/ROS in CORM-2-induced HO-1 expression in HPAEpiCs.

c-Src and EGFR have been shown to regulate the expression of various genes including HO-1 expression [19,52]. Several studies have reported that c-Src stimulates EGFR transactivation via the phosphorylation of cytoplasmic domains of EGFR [41,42]. This was confirmed by our results indicating that CORM-2-induced HO-1 expression was mediated through c-Src and EGFR phosphorylation, which were attenuated by PP1 and AG1478 or transfection with siRNAs. We further investigated the physical association of p47*^phox^*, Nox2, c-Src, and EGFR in CORM-2-mediated ROS production, showing that CORM-2 induced the association between c-Src and EGFR, leading to HO-1 expression. Our results documented that CORM-2 induced the formation of a p47*^phox^*/Nox2/c-Src/EGFR complex that led to HO-1 expression in HPAEpiCs.

PI3K/Akt has been shown to be a downstream component of receptor tyrosine kinases such as EGFR, which are activated by different stimuli [41,53] leading to HO-1 expression [29,54]. In PC12 cells, PI3K/Akt has been documented to mediate HO-1 expression induced by carnosol [55] and NGF [56]. This was confirmed by our observation that indicated that PI3K/Akt played a key role in CORM-2-induced HO-1 expression, which was attenuated by LY294002 and SH-5 or transfection with p110 siRNA. Akt phosphorylation-mediated HO-1 expression by CORM-2 was also attenuated by the inhibitors of PI3K, Akt, c-Src, or EGFR. We found that the involvement of PI3K/Akt in CORM-2-induced HO-1 expression was consistent with the results in cobalt protoporphyrin IX-stimulated Caco-2 cells [57]. Akt has been shown to be a downstream component of ROS in response to high glucose [28], which is consistent with our results that CORM-2-stimulated Akt phosphorylation in HPAEpiCs was attenuated by the inhibitors of Nox and ROS. Taken together, these results indicate that CORM-2-induced HO-1 expression is dependent on PI3K/Akt, which was mediated through Nox2/ROS /c-Src/EGFR signaling in HPAEpiCs.

The activated transcription factors further interacted with the response elements on the HO-1 promoter to regulate gene transcription mediated through several signaling molecules including ROS [32,58]. ROS generation can initiate the HO-1 expression through the degradation of Keap1 and translocation of Nrf2 into the nucleus [59]. ARE1 and ARE2, located on the enhancer regions of HO-1 promoter, play key roles in HO-1 expression exposed to oxidative stresses [60]. The involvement of Nrf2 in these responses was further supported by the results indicating that CORM-2 stimulated Nrf2 accumulation in the nucleus and phosphorylation via Nox/ROS-mediated c-Src/EGFR linking to the PI3K/Akt pathway. Activated Nrf2 binding to AREs on the promoters of HO-1 enhanced the induction of anti-oxidant genes [2]. In our study, the role of Nrf2 in CORM-2-induced HO-1 expression was confirmed by transfection with Nrf2 siRNA. The involvement of AREs in CORM-2-induced HO-1 expression was further supported by the results that CORM-2-stimulated ARE promoter activity was reduced by pretreatment with DPI, APO, NAC, PP1, AG1478, LY294002, or SH-5. These results suggest that CORM-2 induces HO-1 promoter activity via a Nox/ROS-mediated c-Src/EGFR/PI3K/Akt/Nrf2/ARE pathway.

HO-1 is upregulated in response to various insults, oxidative stress, cellular injury, and diseases [2,6,61]. The HO-1 gene is highly regulated by numerous stimulators at the transcriptional regulation in most cell types. A previous report indicated that CORMs can upregulate HO-1 activity and attenuate the LPS-induced inflammatory responses in macrophage cell lines [7] and animal studies [6,36]. Moreover, the overexpression of HO-1 in ovalbumin (OVA)-sensitized guinea pigs effectively decreased inflammatory reaction, mucus secretion, and responsiveness to histamine in airways [62], suggesting that HO-1 exhibits a protecting ability in the host during pulmonary inflammation. In this study, the induction of HO-1 by CORM-2 protected against TNF-α-induced ICAM-1 expression and leukocytes infiltration in in vitro and in vivo studies. Importantly, previous reports have also indicated that CORM-2-derived CO release can attenuate cell sequestration, nuclear factor kappa B (NF-κB) activity, and ICAM-1 expression of leukocytes after lung injury [63], as well as regulate the expressions of surface adhesion molecules on human umbilical vein endothelia cells to affect leukocyte attachment [64]. Our previous report also indicated that the induction of HO-1 protein by CoPPIX inhibited TNF-α-induced ICAM-1 and VCAM-1 expression, which is revered by ZnPPIX (an inhibitor of HO-1) in HTSMCs [12]. Therefore, the upregulation of HO-1 exhibited benefits to prevent airway inflammation in the lung.

In the respiratory system, the pulmonary alveolar epithelium is composed of type I and type II alveolar epithelial cells. Type I cells comprise ≈40% of the alveolar epithelium and form the epithelial component of the thin air–blood barrier and are responsible for the gas exchange of CO_2_/O_2_ that takes place. Type II cells comprise 60% of the alveolar epithelium and 15% of the peripheral lung cells. Type II cells exert the most important function to secrete materials to support surface tension, clean inhaled particles, such as bacteria, and prevent interstitial fluid into the alveolus [65]. The biological activity of the primary human pulmonary type II cells producing surfactant protein (SP)-C, cytokines, and intercellular adhesion molecule-1 is vigorous in response to stimulation with TNF-α [65]. In our study, HPAEpiCs contained types I and II, which are not recommended for expanding or long-term cultures, because the cells differentiate to become type I cells immediately after plating. Type I alveolar epithelial cells do not proliferate in culture. Thus, only the passages of HPAEpiCs from 4 to 6 were used throughout this study.

## 5. Conclusions

The present study confirmed that CORM-2-induced HO-1 expression is mediated through the Nox/ROS- and c-Src/EGFR-dependent activation of PI3K/Akt, linked to the upregulation of Nrf2/AREs, which promote HO-1 expression and enzymatic activity in HPAEpiCs. Based on observations from the literature and our findings, Figure 8 depicts a model for the molecular mechanisms underlying CORM-2-induced HO-1 expression and activity in HPAEpiCs. The results obtained with cellular and animal experiments indicate that CORM-2 not only induces HO-1 to attenuate the inflammatory response, but also provides a therapeutic strategy for airway inflammatory disorders.
